# Comparison of different methods of obturator nerve block in transurethral resection of bladder tumors: A systematic review and network meta‐analysis

**DOI:** 10.1002/cam4.5364

**Published:** 2022-11-07

**Authors:** Jinhao Wu, Yafen Gao, Zhiyong Xiong, Xiong Xiao, Jun Yang, Xiong Yang, Yu Huang

**Affiliations:** ^1^ Department of Urology, Union Hospital, Tongji Medical College Huazhong University of Science and Technology Wuhan China; ^2^ Department of Anesthesiology, Union Hospital, Tongji Medical College Huazhong University of Science and Technology Wuhan China

**Keywords:** bladder cancer, lumbar anesthesia, obturator nerve block, transurethral resection of bladder tumor

## Abstract

**Background:**

Bladder cancer is the most common malignancy of the urinary system, and accounts for 3% of newly diagnosed tumors. Transurethral resection of bladder tumor plays a key role in treating bladder cancer, among which one of the most serious complications is bladder perforation caused by obturator nerve reflex. Obturator nerve reflex can be prevented by inducing obturator nerve block after lumbar anesthesia. However, No study so far has compared the inhibitory effect of different obturator nerve block approaches on intraoperative obturator nerve reflex and bladder perforation.

**Method:**

In this study, we conducted a network meta‐analysis (NMA) of studies comparing the efficacy of different obturator nerve block approaches performed after lumbar anesthesia in operation.

**Result:**

The distal obturator nerve block guided by peripheral nerve stimulator is the best approach for preventing obturator reflex. The proximal obturator nerve block guided by ultrasound is the best approach for preventing bladder perforation.

**Conclusion:**

Spinal anesthesia combined with the distal obturator nerve block guided by peripheral nerve stimulator is the most optimal approach to prevent the obturator nerve reflex. But the doctor should choose the appropriate anesthesia method according to the patient's general condition, tumor location, and doctor's proficiency in puncture techniques.

## 1 INTRODUCTION

Bladder cancer is the most common malignancy of the urinary system, and accounts for 3% of newly diagnosed tumors and 2.1% of all cancer‐related deaths worldwide.^1^ Transurethral resection of bladder tumor (TURBT) remains the cornerstone of bladder cancer diagnosis and therapy, and is recommended by the European Association of Urology (EAU), National Comprehensive Cancer Network (NCCN), and American Urological Association (AUA). Pathological examination of the tissues resected by TURBT can help assess the depth of bladder cancer invasion, which is critical for tumor staging. Furthermore, the non‐muscle invasive bladder tumors can be removed to the maximum extent by TURBT in combination with intravesical chemotherapy.^2^

However, intraoperative complications of TURBT such as bladder perforation and hemorrhage are closely related to relatively high rates of postoperative recurrence and progression. Bladder perforation increases the risk of intraoperative trans‐urethral resection prostate syndrome, hemorrhage, and the possibility of transferring to open surgery. It may also lead to delay intravesical chemotherapy, resulting in worse prognosis.^3^ Meanwhile, avoiding bladder perforation may lead to insufficient depth of removal. The higher recurrence rate after TURBT is often attributed to the incomplete removal of the bladder tumor. As per the guidelines of the EAU, NCCN, and AUA, complete tumor resection during TURBT is considered as such when only the detrusor muscle tissue remains in the bladder,^2^ and can dramatically reduce the short‐term recurrence after TURBT. However, studies show that only 52% of the post‐TURBT samples contain the detrusor muscle tissue.^4^ In addition, the fractioned technique of TURBT can aid in the engraftment of tumor cells into healthy bladder mucosa, thereby increasing the chances of recurrence.^5^ Therefore, a proper depth of resection is critical when performing TURBT to not only ensure complete tumor removal but also avoid bladder perforation that may occur if the resection is performed too deep. Reducing the incidence of intraoperative complications during TURBT can improve the prognosis of bladder cancer patients.

The obturator nerve is located near the inferolateral wall of the bladder. It originates from the 2nd, 3rd, and 4th lumbar nerve roots, runs toward the adductor muscle, and enters the pelvis near the sacroiliac joint.^6^ Resection of bladder tumors at the lateral bladder wall stimulates the obturator nerve and results in adductor muscle contraction, a phenomenon known as the obturator nerve reflex.^7^ The latter significantly increases the likelihood of intraoperative bleeding and vesical perforation during TURBT, resulting in incomplete tumor resection or extravesical spread of the tumor, eventually worsening patient prognosis. Various strategies are followed to avoid the obturator nerve reflex, including the administration of systemic neuromuscular blocking agents, decreasing the current used for resection, and selective obturator nerve blocking (ONB).^6^ Since most TURBT operations are performed under spinal anesthesia, the obturator nerve reflex can be prevented by inducing ONB after lumbar anesthesia.

The various approaches currently used for ONB differ in terms of the injection sites and methods, the scope of blocking, and the instruments. Based on the injection site, ONB can be performed using the proximal, distal, or transvesical approach. The block could be guided by a peripheral nerve stimulator or ultrasound. Muscle contraction caused by peripheral nerve stimulator lead to a more precise nerve location, and ultrasound can help to observe the injection site and range. The combination of both can possibly further increase the success rate since the contraction of adductor muscles can be confirmed with ultrasound guidance during nerve stimulation. No study so far has compared the inhibitory effect of different ONB methods on the intraoperative obturator nerve reflex and bladder perforation. To this end, we conducted a network meta‐analysis (NMA) of studies comparing the efficacy of various ONB approaches performed after lumbar anesthesia. The study followed the PRISMA guidelines (see Appendix S1 for the checklist).

## 2 METHODS

### 2.1 Literature search strategy and screening criteria

The PUBMED, Cochrane, and EMBASE databases were searched for all randomized controlled trials (RCTs) and retrospective studies on TURBT performed with ONB under lumbar anesthesia using any of the following methods: proximal ONB guided by a peripheral nerve stimulator (pONB NS), proximal ONB guided by ultrasound (pONB US), distal ONB guided by a peripheral nerve stimulator (dONB NS), distal ONB guided by ultrasound (dONB US), distal ONB guided by ultrasound and a peripheral nerve stimulator (dONB NS + US), transvesical ONB (tONB), and transvesical ONB guided by a cystoscope needle electrode (tONB NS).

Two authors searched the databases for articles published till March 30, 2002, using keywords including bladder cancer, transurethral resection of bladder tumor, lumbar anesthesia, and obturator nerve block. Both MeSH terms and free texts were used to retrieve the studies. The references in the included articles were also manually searched for additional studies. For studies with multiple publications, only the reports with complete data were selected.

### 2.2 Outcome selection and data extraction

The following data were extracted from the selected studies: the total number of cases, number of cases with the final outcome, incidence of obturator nerve reflex, frequency of bladder perforation, tumor location, and other information. The primary outcomes were adductor jerking and bladder perforation caused by obturator reflex during TURBT. The incidences of obturator nerve reflex and bladder perforation during TURBT with the different ONB methods were compared.

### 2.3 Quality assessment

The risk of bias in the RCTs was evaluated using Review Manager version 5.4.0 (Cochrane Collaboration, Oxford, UK), and included selection bias, performance bias, detection bias, attrition bias, reporting bias, and other bias. Newcastle‐Ottawa scale (NOS) was used to evaluate the retrospective cohort studies. The online tool Confidence in Network Meta‐Analysis (CINeMA, https://cinema.ispm.unibe.ch/) was used to evaluate the results of the NMA.

### 2.4 Data analysis

Stata 16.0 software (Stata Corp LP) and R Studio 4.1.3 software were used for data analysis. Markov Chain Monte Carlo (MCMC) method was used for NMA with Bayesian framework. Four of the chains were used for simulation and the number of iterations was set to 50,000. In addition, the stringency of the results was evaluated by Potential Scale Reduction Factor (PSRF). The PRSF values ranging from 1 to 1.05 are indicative of good stringency and high reliability. The Node splitting method was used to measure inconsistency. In case of no obvious inconsistency, the consistency model was used, otherwise, the inconsistency model was used. The efficacy of the different ONB methods was analyzed by plotting ranking curves and cumulative ranking curves and compared by calculating the odds ratio (OR) and 95% confidence interval (CI).

## 3 RESULTS

### 3.1 Characteristics of the included studies

**FIGURE 1:**
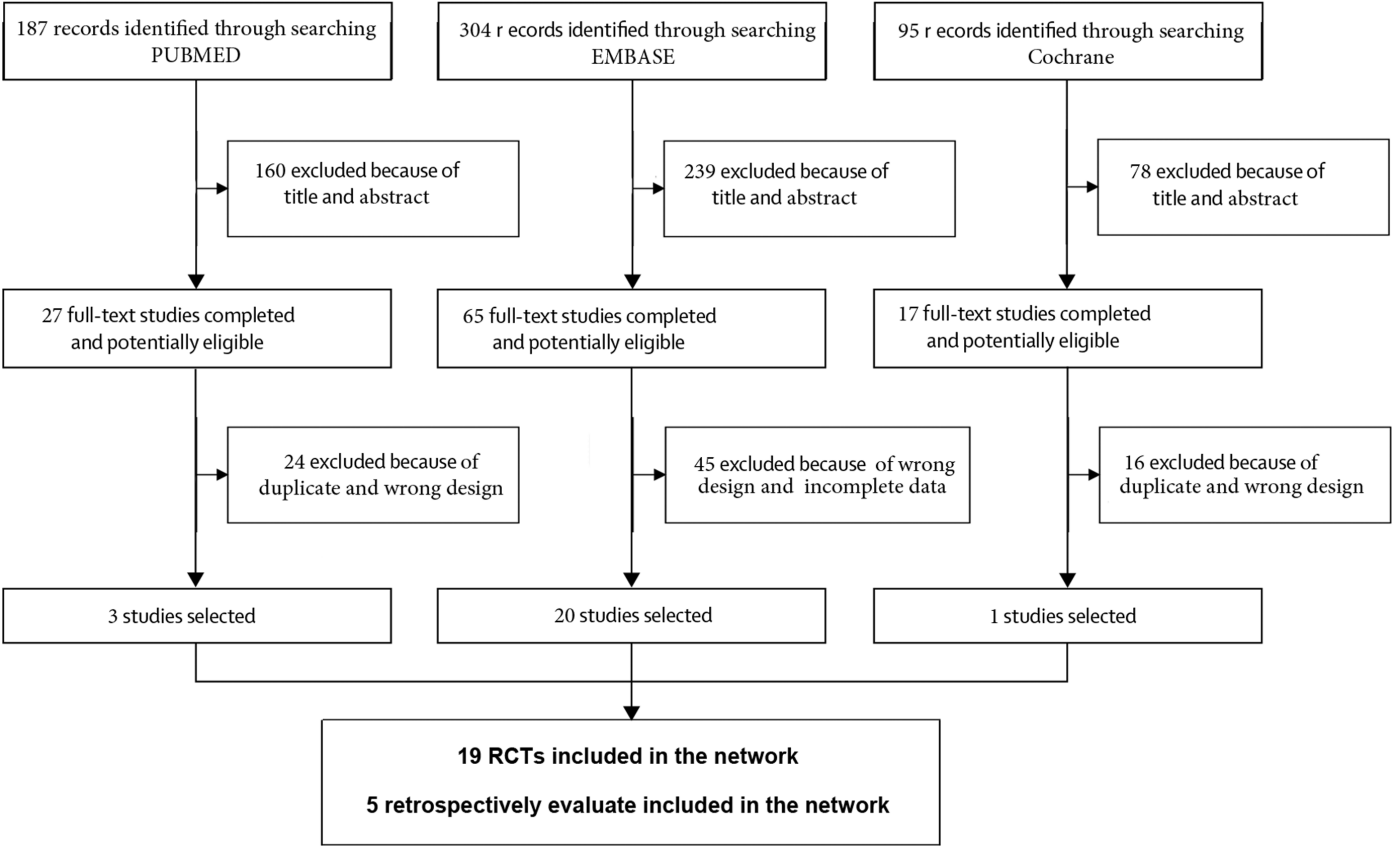
Flow diagram of the search process for eligible studies.

Based on the inclusion and exclusion criteria, 24 studies were included in the NMA.^8^, ^9^, ^10^, ^11^, ^12^, ^13^, ^14^, ^15^, ^16^, ^17^, ^18^, ^19^, ^20^, ^21^, ^22^, ^23^, ^24^, ^25^, ^26^, ^27^, ^28^, ^29^, ^30^, ^31^ The literature screening process is outlined in Figure 1. There were 19 RCTs and 5 retrospective cohort studies, including 1 three‐arm study. The studies compared seven different ONB methods and included 1722 independent participants. The details are summarized in Table 1. The study participants were classified into the following groups: (a) lumbar anesthesia (control group), (b) lumbar anesthesia with proximal ONB guided by peripheral nerve stimulator (pONB NS), (c) distal ONB guided by ultrasound and a peripheral nerve stimulator (dONB NS + US), (d) transvesical ONB guided by a cystoscope needle electrode (tONB NS), (e) transvesical ONB (tONB), (f) distal ONB guided by ultrasound (dONB US), (g) proximal ONB guided by ultrasound (pONB US), and (h) distal ONB guided by a peripheral nerve stimulator (dONB NS) as shown in Appendix S4. Most participants were in the control group. The most common procedure was pONB NS (11/24 studies), followed by dONB NS + US (9/24), dONB US (6/24), dONB NS (4/24), tONB (3/24), tONB NS (2/24), and pONB US (2/24).

**TABLE 1:** Basic features of included studies

	Study name	Type	Number	Age	Dose	Endpoint	Study design	Tumor position
1	Cyrus Emir Alavi 2017^8^	A	15	67	‐	Adductor muscle jerking	RCT	Lateral wall
		B	15	69.2	15 ml 1% lidocaine			
2	Guven Erbay 2016^9^	A	49	69.2	‐	Adductor muscle jerking	Retrospectively evaluate	Lateral wall
		C	47	69.6	10 ml 1% lidocaine			
3	Zeki Tuncel Tekgül 2013^10^	A	36	65.8	‐	Tumor recurrence	Retrospectively evaluate	Lateral wall
		B	32	67.1	10 ml 0.25% levobupivacaine			
4	Deniz Bolat 2015^11^	A	35	67.7	‐	Adductor muscle jerking	RCT	Lateral wall
		B	35	70.1	10 ml 0.25% levobupivacaine			
5	Mohammad Hatef Khorrami 2010^12^	A	30	61	‐	Leg jerking	RCT	Lateral wall
		D	30	62	10 ml 1% lidocaine			
6	Kamil DARÇIN 2012^13^	A	30	61.1	‐	Adductor muscle jerking	RCT	Lateral wall
		B	30	60.9	10 ml 3.75% levobupivacaine			
7	Deepak Sharma 2017^14^	B	20	66.4	10 ml 2% lignocaine+5 ml 0.5% bupivacaine	Resectability of tumor	RCT	Lateral wall
		E	20	63.6				
8	Chao Han 2019^15^	F	25	65.1	10 ml 0.5% bupivacaine	Adductor muscle jerking	RCT	Lateral wall
		G	25	64.1				
9	Srilata Moningi 2014^16^	B	30	58.6	10 ml 0.25% bupivacaine	Adductor muscle jerking	RCT	Lateral wall
		H	30	58.6				
10	Recai Dagli 2019^17^	B	28	61	10 ml 0.25% bupivacaine	Duration of the procedure	RCT	Lateral wall
		H	33	58.6				
11	Youn Yi Jo 2011^18^	B	50	64.5	10 ml 1% lidocaine	Success rate	RCT	Lateral wall
		H	51	62.4				
12	Dawood Agha mohammadi 2017^19^	H	35	63.23	10 ml 1% lidocaine	Success rate	RCT	Lateral wall
		B	35	67.49				
13	Yong Beom Kim 2019^20^	C	31	70	10 ml 1.5% lidocaine	Failure rate	RCT	Not mentioned
		F	31	68				
14	Alberto Manassero 2012^21^	F	25	70	10 ml 2% lidocaine	Duration of the procedure	RCT	Lateral wall
		C	25	66.5				
15	Nida Farooq Shah 2017^22^	F	30	53.87	10 ml 0.5% bupivacaine	Success rate	RCT	Lateral wall
		C	30	55.57				
16	Mohammadhatef Khorrami 2012^23^	D	34	55.4	15 ml 2% lidocaine	Leg jerking	RCT	Lateral wall
		A	31	57.8	15 ml physiologic saline			
		E	47	58.4	15 ml 2% lidocaine			
17	Cuneyd Sevinc 2021^24^	A	30	64.3	‐	Adductor muscle jerking	Retrospectively evaluate	Lateral wall
		B	30	65.1	5 ml 2% prilocaine			
18	Nici Markus Dreger 2021^25^	A	58	71.8	‐	Adductor muscle jerking	Retrospectively evaluate	Lateral wall
		E	36	70.9	10–20 ml 2% lidocaine			
19	Omer Gokhan Doluoglu 2021^26^	A	53	64.3	‐	Tumor recurrence	RCT	Lateral wall
		C	51	64.6	10 ml 1% lidocaine			
20	Gamal E. Abourjila 2020^27^	A	39	61.3	‐	Adductor muscle jerking	RCT	Lateral wall
		C	35	65.2	10 ml 1% lidocaine			
21	Dr.S. Narmatha Yangtse 2020^28^	F	30	54.7	10 ml 0.5% bupivacaine	Success rate	RCT	Lateral wall
		C	30	53.6				
22	Brijesh Tiwari 2022^29^	C	25	65	10 ml 1.5% lidocaine	Success rate	RCT	Not mentioned
		F	25	67				
23	Houman Teymourian1 2018^30^	B	62	63.21	10 ml 1.5% lidocaine	Adductor muscle jerking	RCT	Not mentioned
		G	62	62.11				
24	Ozan Horsanali 2021^31^	A	55	66.02	‐	Tumor recurrence	Retrospectively evaluate	Lateral wall
		C	51	63.29	10 ml 2% lidocaine			

Abbreviation: RCT, randomized controlled trial.

### 3.2 Quality assessment

**FIGURE 2:**
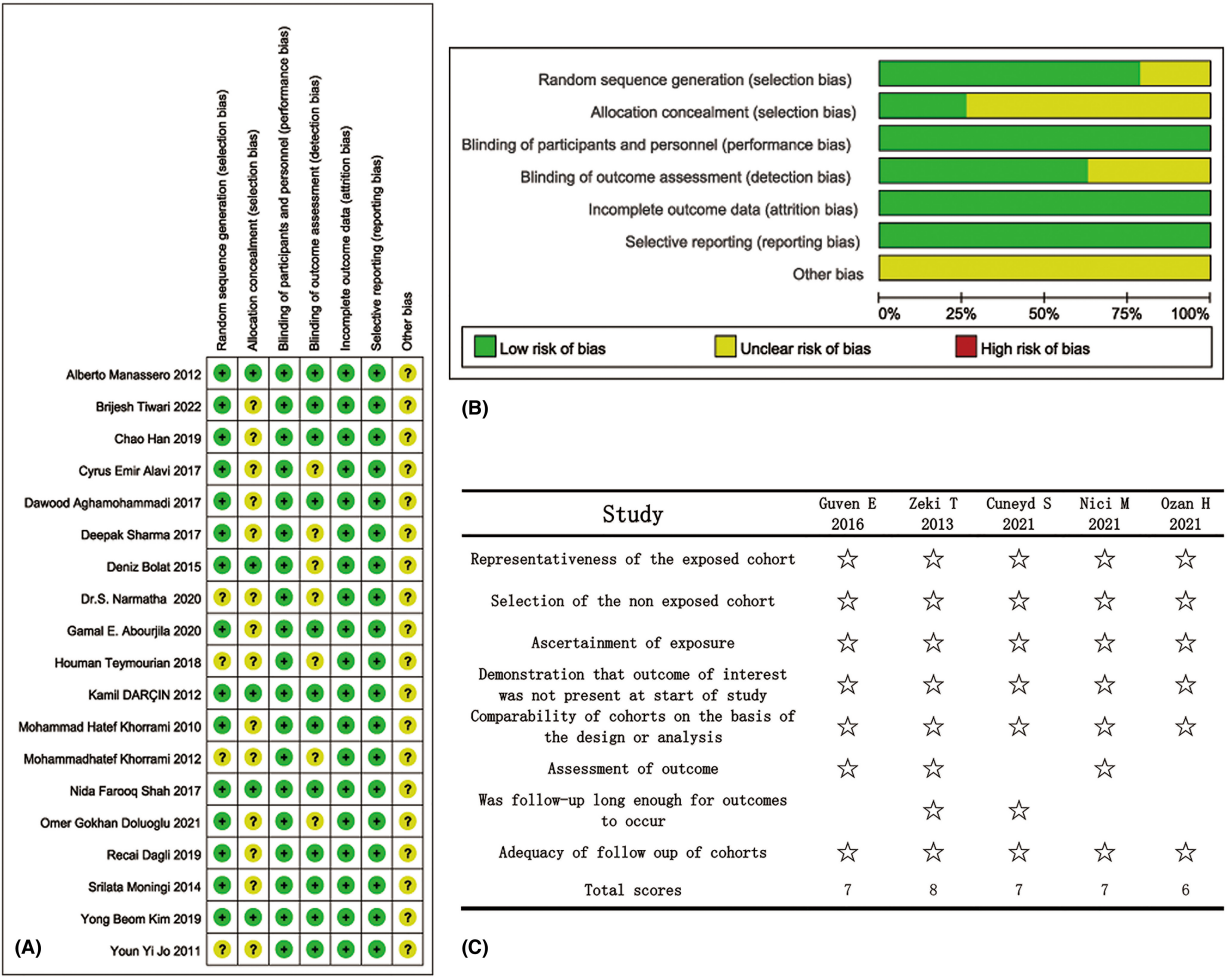
Quality assessments (A and B: risk of bias, C: assessments of confidence).

The results of the risk assessment for the 19 RCTs are shown in Figure 2A,B. Four studies did not provide details regarding the randomization procedures, and only five reports described allocation concealment. In seven studies, it was unclear if the outcomes were analyzed in a blinded manner. The risk assessment of bias in the five retrospective cohort studies is shown in Figure 2C. CINeMA showed high to low confidence for intraoperative obturator nerve reflex, primarily due to intra‐study bias and imprecision (Table 2). For bladder perforation, the confidence was low to very low due to the same reasons (Table 3).

**TABLE 2:** Certainty of evidence evaluated with CINeMA for intraoperative obturator nerve reflex

Comparison	Study No. and design	Within‐study bias	Reporting bias	Indirectness	Imprecision	Heterogeneity	Incoherence	GRADE
Mixed evidence								
A vs. B	3 RCTs 1 Retrospective	Some concerns	Low risk	No concerns	No concerns	No concerns	No concerns	Moderate
A vs. C	3 RCTs 1 Retrospective	Some concerns	Low risk	No concerns	No concerns	No concerns	No concerns	Moderate
A vs. D	2 RCTs	Some concerns	Low risk	No concerns	No concerns	No concerns	No concerns	Moderate
A vs. E	1 RCT 1 Retrospective	Some concerns	Low risk	No concerns	No concerns	No concerns	No concerns	Moderate
B vs. E	1 RCT	Some concerns	Low risk	No concerns	Major concerns	No concerns	No concerns	Low
B vs. G	1 RCT	Major concerns	Low risk	No concerns	No concerns	No concerns	No concerns	Low
B vs. H	4 RCTs	Some concerns	Low risk	No concerns	Major concerns	No concerns	No concerns	Low
C vs. F	5 RCTs	No concerns	Low risk	No concerns	No concerns	No concerns	No concerns	High
D vs. E	1 RCT	Some concerns	Low risk	No concerns	Major concerns	No concerns	No concerns	Low
F vs. G	1 RCT	Some concerns	Low risk	No concerns	No concerns	No concerns	No concerns	Moderate
Indirect evidence								
A vs. F	–	Some concerns	Low risk	No concerns	No concerns	No concerns	No concerns	Moderate
A vs. G	–	Some concerns	Low risk	No concerns	No concerns	No concerns	No concerns	Moderate
A vs. H	–	Some concerns	Low risk	No concerns	No concerns	No concerns	No concerns	Moderate
B vs. C	–	Some concerns	Low risk	No concerns	Major concerns	No concerns	No concerns	Low
B vs. D	–	Some concerns	Low risk	No concerns	Major concerns	No concerns	No concerns	Low
B vs. F	–	Some concerns	Low risk	No concerns	No concerns	Major concerns	No concerns	Low
C vs. D	–	Some concerns	Low risk	No concerns	Major concerns	No concerns	No concerns	Low
C vs. E	–	Some concerns	Low risk	No concerns	Major concerns	No concerns	No concerns	Low
C vs. G	–	Some concerns	Low risk	No concerns	Major concerns	No concerns	No concerns	Low
C vs. H	–	Some concerns	Low risk	No concerns	Major concerns	No concerns	No concerns	Low
D vs. F	–	Some concerns	Low risk	No concerns	Major concerns	No concerns	No concerns	Low
D vs. G	–	Some concerns	Low risk	No concerns	Major concerns	No concerns	No concerns	Low
D vs. H	–	Some concerns	Low risk	No concerns	Major concerns	No concerns	No concerns	Low
E vs. F	–	Some concerns	Low risk	No concerns	Major concerns	No concerns	No concerns	Low
E vs. G	–	Some concerns	Low risk	No concerns	No concerns	No concerns	No concerns	Moderate
E vs. H	–	Some concerns	Low risk	No concerns	Major concerns	No concerns	No concerns	Low
F vs. H	–	Some concerns	Low risk	No concerns	No concerns	No concerns	No concerns	Moderate
G vs. H	–	Some concerns	Low risk	No concerns	Major concerns	No concerns	No concerns	Low

Abbreviations: CINeMA, confidence in network meta‐analysis; GRADE, grade of recommendations assessment, development, and evaluation; RCT, randomized controlled trial.

**TABLE 3:** Certainty of evidence evaluated with CINeMA for bladder perforation

Comparison	Study No. and design	Within‐study bias	Reporting bias	Indirectness	Imprecision	Heterogeneity	Incoherence	GRADE
Mixed evidence								
A vs. B	1 RCTs 2 Retrospective	Some concerns	Low risk	No concerns	No concerns	Major concerns	No concerns	Low
A vs. C	1 RCTs 1 Retrospective	Some concerns	Low risk	No concerns	Major concerns	No concerns	No concerns	Low
A vs. E	1 Retrospective	Major concerns	Low risk	No concerns	Major concerns	No concerns	No concerns	Very low
B vs. E	1 RCT	Some concerns	Low risk	No concerns	Major concerns	No concerns	No concerns	Low
B vs. G	1 RCT	Major concerns	Low risk	No concerns	No concerns	Major concerns	No concerns	Very low
Indirect evidence								
A vs. G	–	Major concerns	Low risk	No concerns	No concerns	No concerns	No concerns	Low
B vs. C	–	Some concerns	Low risk	No concerns	Major concerns	No concerns	No concerns	Low
C vs. E	–	Some concerns	Low risk	No concerns	Major concerns	No concerns	No concerns	Low
C vs. G	–	Some concerns	Low risk	No concerns	Major concerns	No concerns	No concerns	Low
E vs. G	–	Major concerns	Low risk	No concerns	Major concerns	No concerns	No concerns	Very low

Abbreviations: CINeMA, confidence in network meta‐analysis; GRADE, grade of recommendations assessment, development, and evaluation; RCT, randomized controlled trial.

### 3.3 Network diagram

**FIGURE 3:**
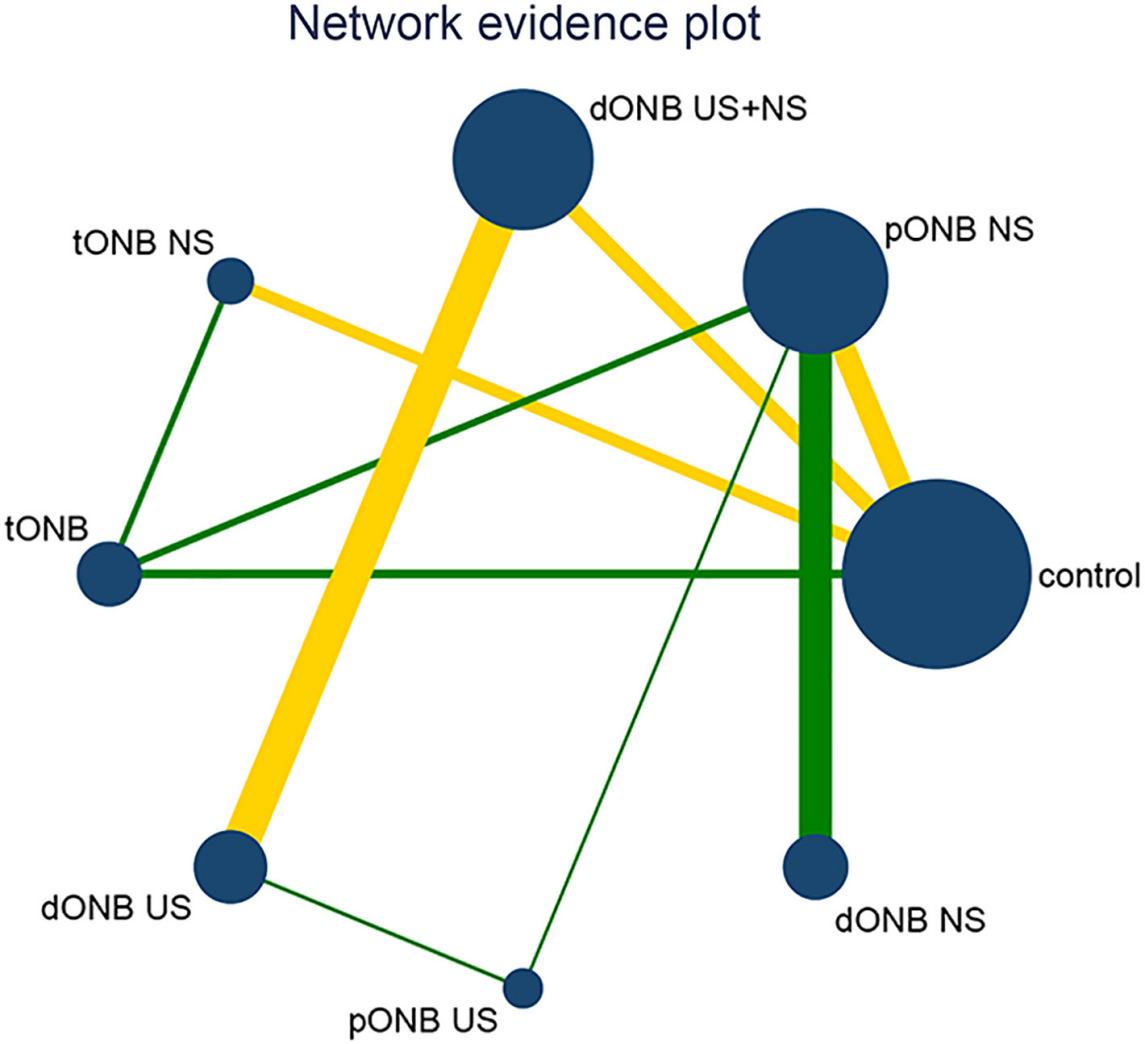
Network plot for intraoperative obturator nerve reflex.

**FIGURE 4:**
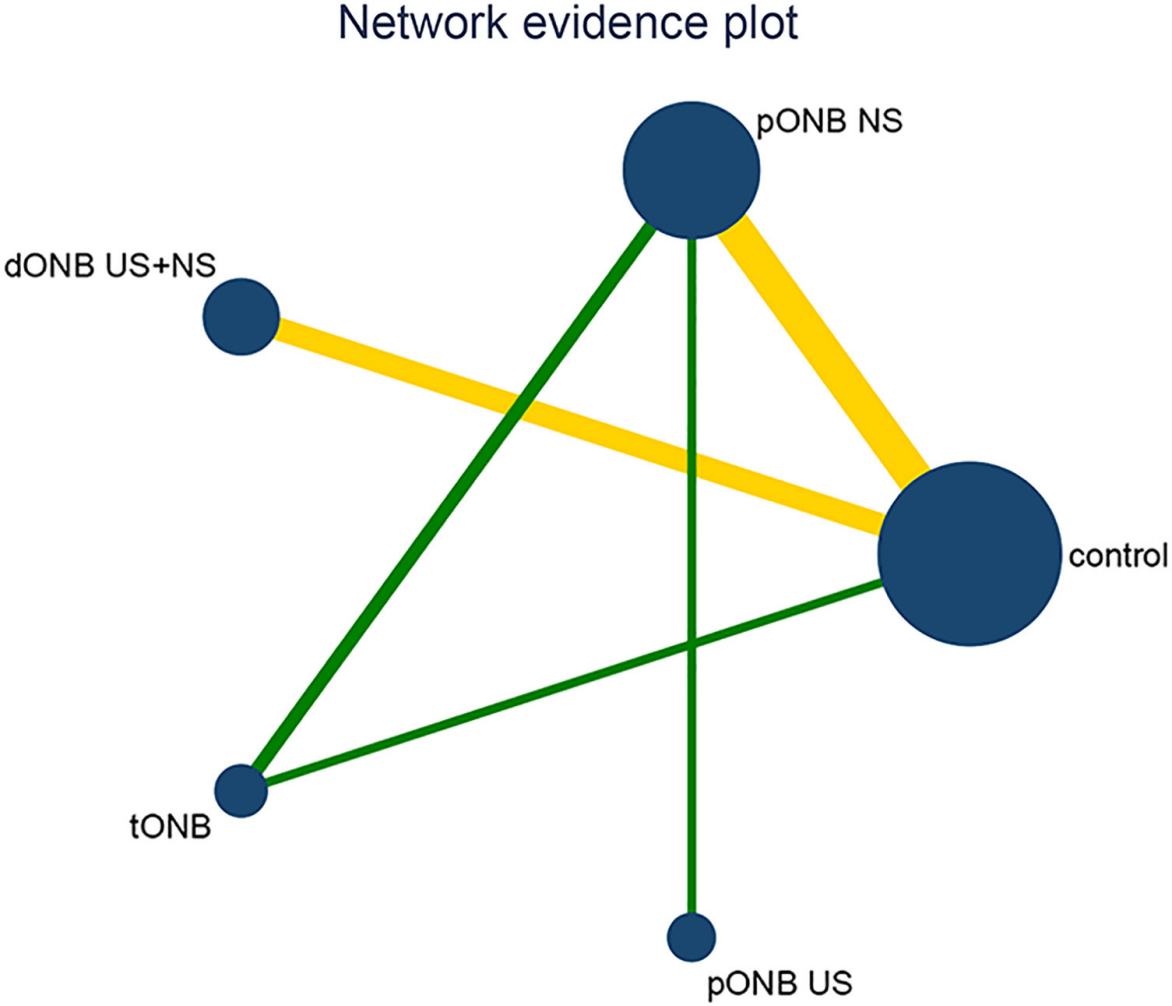
Network plot for bladder perforation.

**FIGURE 5:**
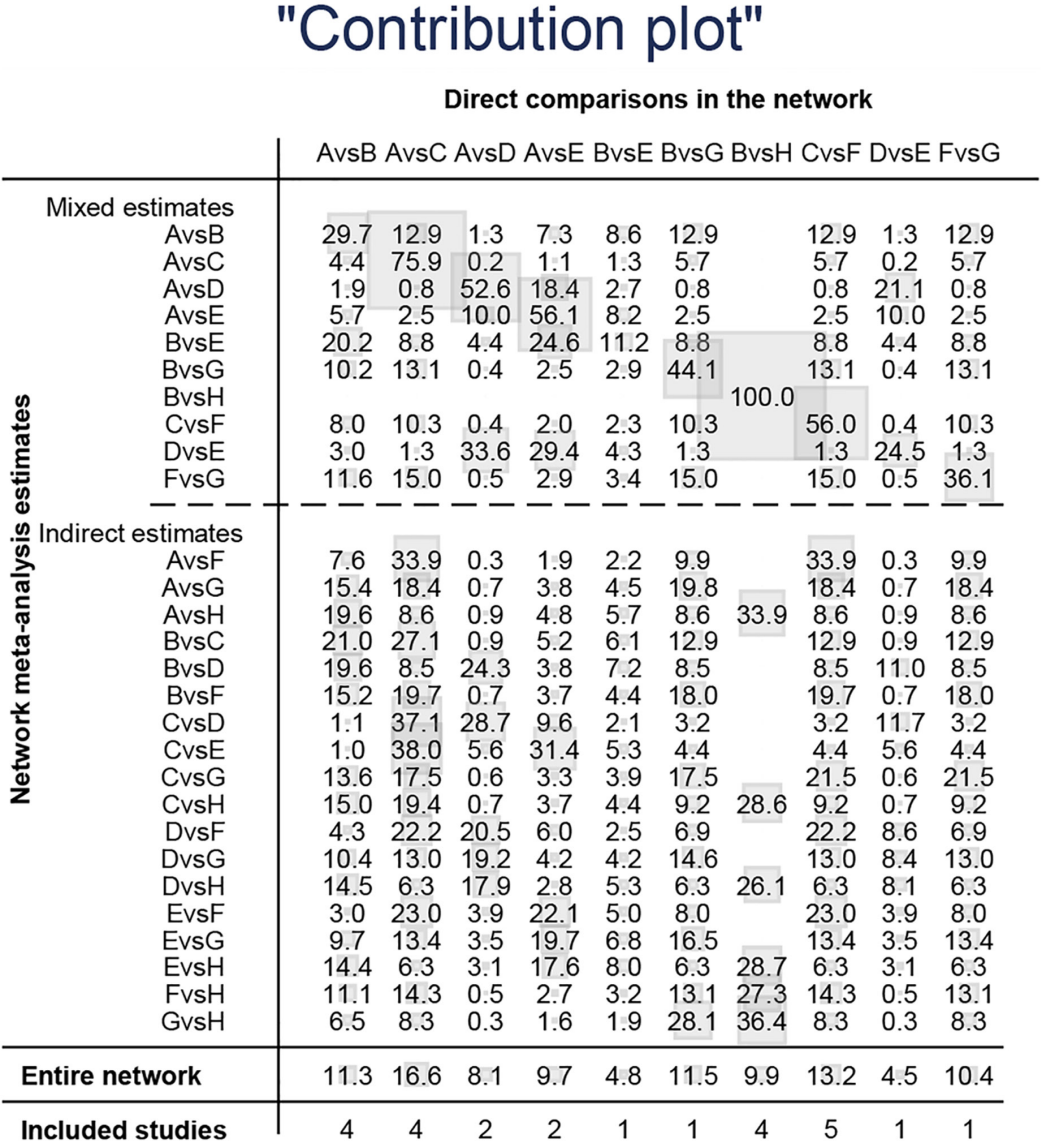
Contribution plot for intraoperative obturator nerve reflex.

**FIGURE 6:**
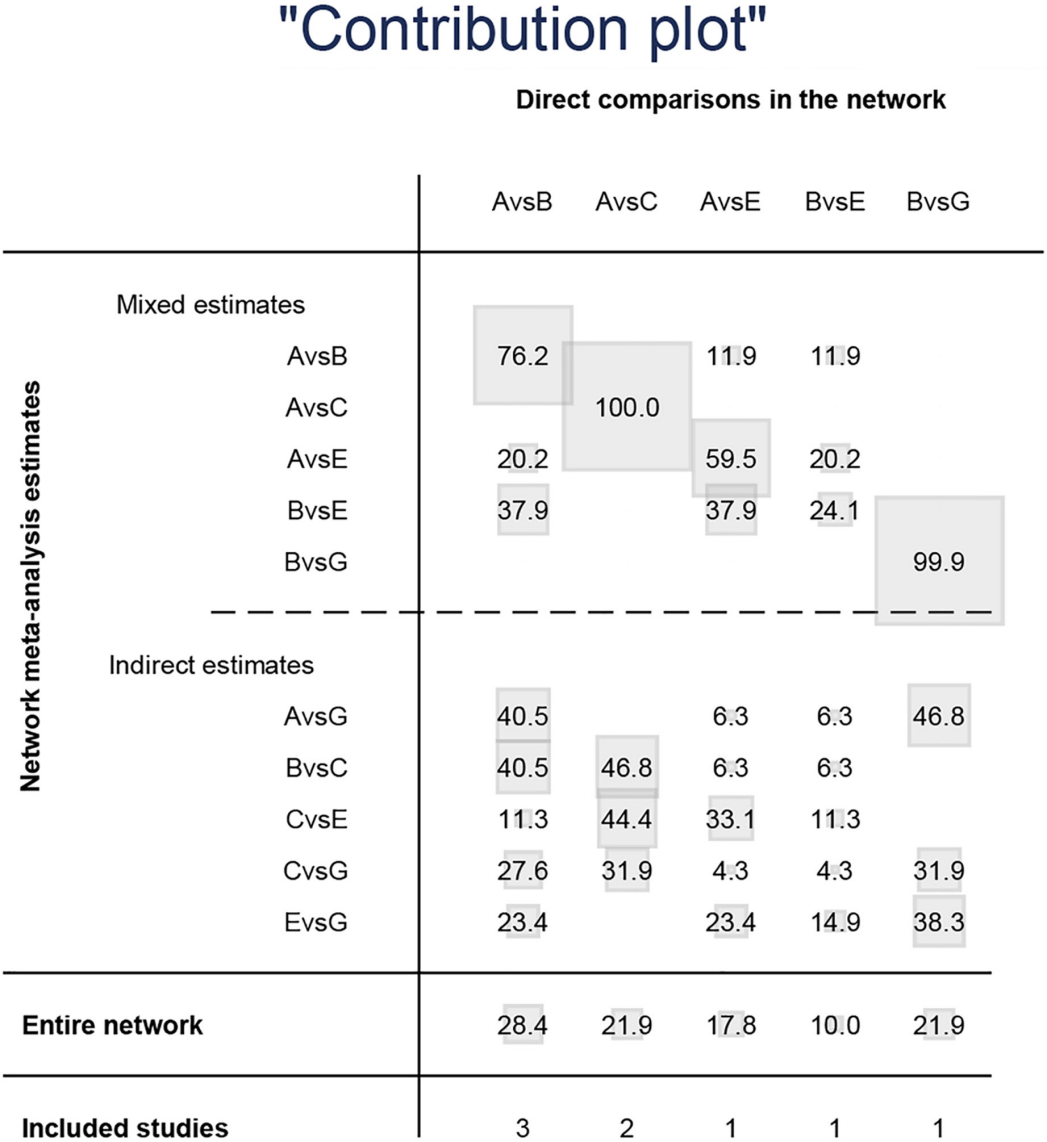
Contribution plot for bladder perforation.

Twenty‐three studies reported the incidence of intraoperative obturator nerve reflex during TURBT, involving six methods of ONB. Eight studies reported the rates of bladder perforation during TURBT, involving five methods of ONB. The network diagrams of the different ONB methods are shown in Figures 3 and 4. Each circle represents a treatment method, and their size reflects the number of trials. Direct comparisons between any two treatments are indicated with a line linking the circles, and the thickness of the line reflects the sample size. The contribution of each group is shown in Figures 5 and 6.

### 3.4 Stringency and inconsistency evaluation

**FIGURE 7:**
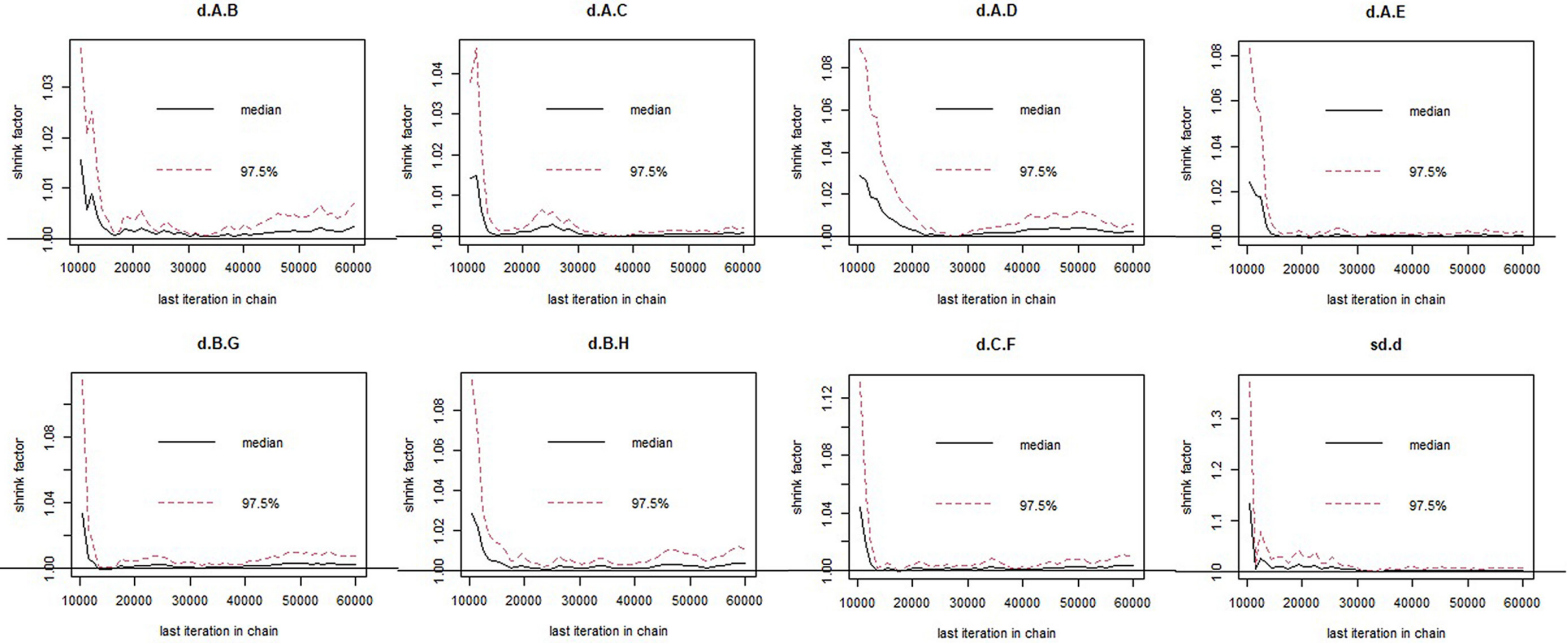
Evaluation of Astringency for intraoperative obturator nerve reflex.

**FIGURE 8:**
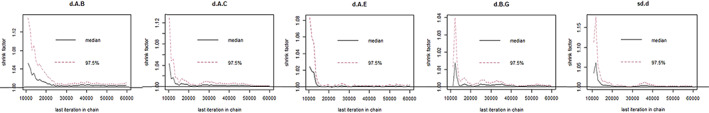
Evaluation of Astringency for bladder perforation.

**FIGURE 9:**
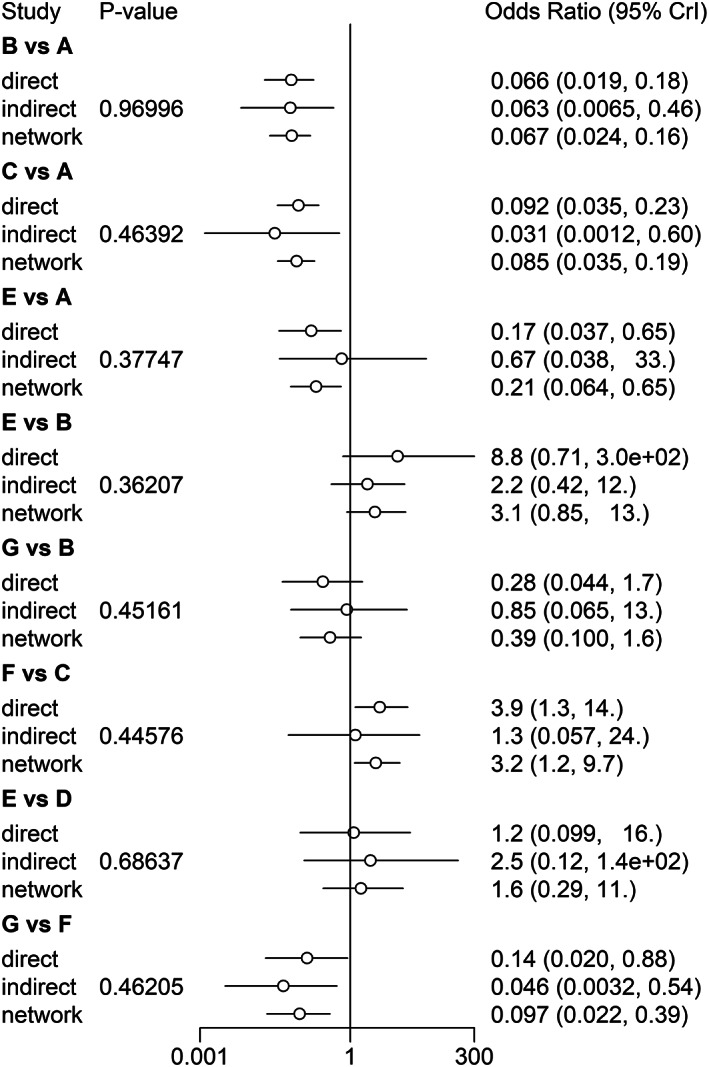
Evaluation of Inconsistency for intraoperative obturator nerve reflex.

**FIGURE 10:**
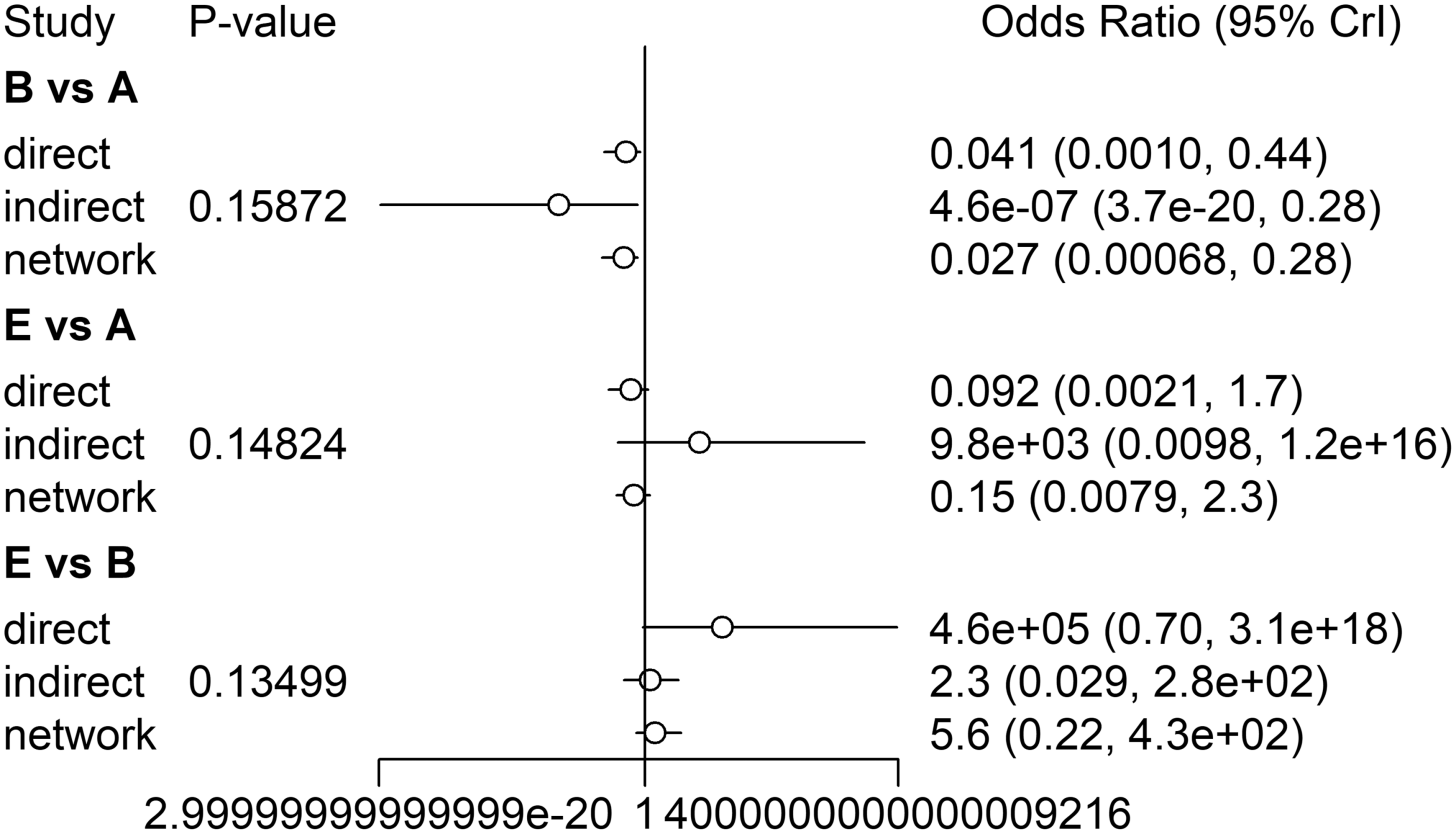
Evaluation of Inconsistency for bladder perforation.

The stringency of the results is summarized in Figures 7 and 8. The PSRF were all close to 1, and the maximum was less than 1.005, indicating satisfactory stringency. Therefore, the Bayes model constructed in this study can effectively predict the data. In addition, the node splitting method indicated that the inconsistency among the studies included in the analysis was not significant (*p* ≤ 0.05; Figures 9 and 10). Given the lack of a significant difference between direct comparison and indirect comparison, a consistency model was used. Finally, there was no obvious heterogeneity in the test results (Appendices S2 and S3).

### 3.5 Obturator nerve reflex with different ONB methods

**FIGURE 11:**
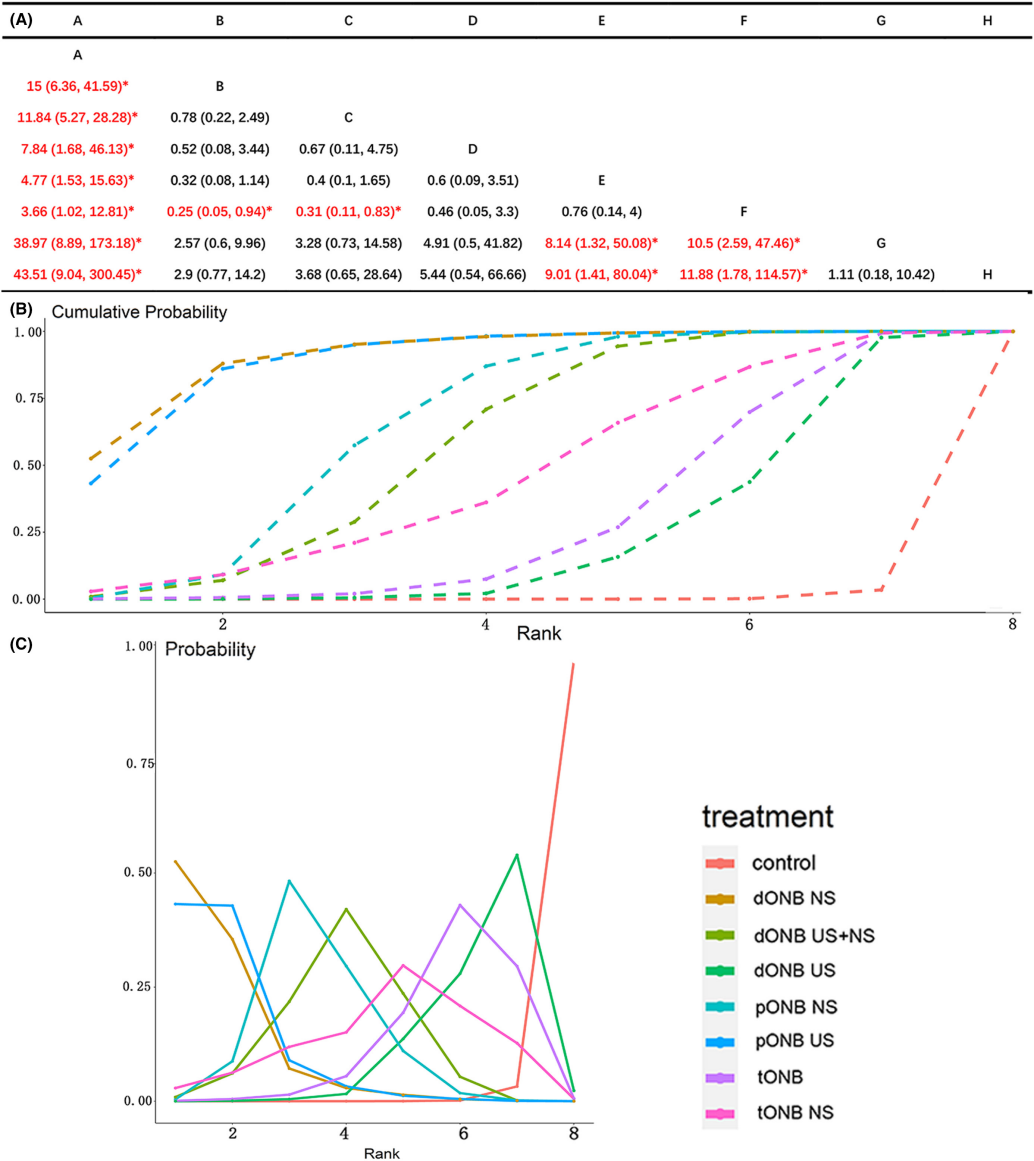
NMA result for intraoperative obturator nerve reflex (A: result of pair‐compare, B: cumulative probability ranking chart, C: probability ranking chart).

Twenty‐three studies reported the incidence of obturator nerve reflex during TURBT, involving eight methods of ONB. The results of NMA showed that the different ONB methods inhibited obturator nerve reflex compared to that in the untreated control group. The therapeutic effects of dONB NS, pONB US, pONB NS, and dONB NS + US were superior to that of dONB US, whereas PONB US, and dONB NS exhibited better effects compared to tONB (*p* < 0.05). Comparisons between other interventions did not yield significant results (*p* > 0.05; Figure 11A). Rank probability analysis with the consistency model further indicated that dONB NS is the best approach for preventing obturator reflex. The probability ranking chart and the cumulative probability ranking chart are shown in Figure 11B,C, respectively.

### 3.6 Bladder perforation with different ONB methods

**FIGURE 12:**
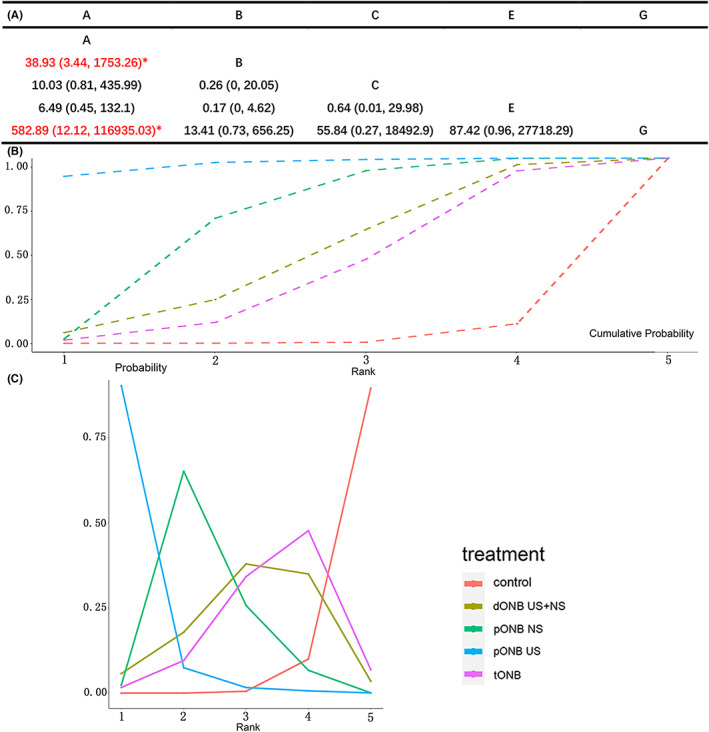
NMA result for bladder perforation (A: result of pair‐compare, B: cumulative probability ranking chart, C: probability ranking chart).

Eight studies reported the incidence of bladder perforation during TURBT, involving five ONB methods. Results of the NMA showed that both pONB NS and pONB US are superior to lumbar anesthesia alone (*p* ≤ 0.05; Figure 12A). Rank probability analysis showed that pONB US is the most likely to prevent bladder perforation. The probability ranking chart and the cumulative probability ranking chart are shown in Figures 12B,C, respectively.

### 3.7 Comparison‐adjusted funnel plot

**FIGURE 13:**
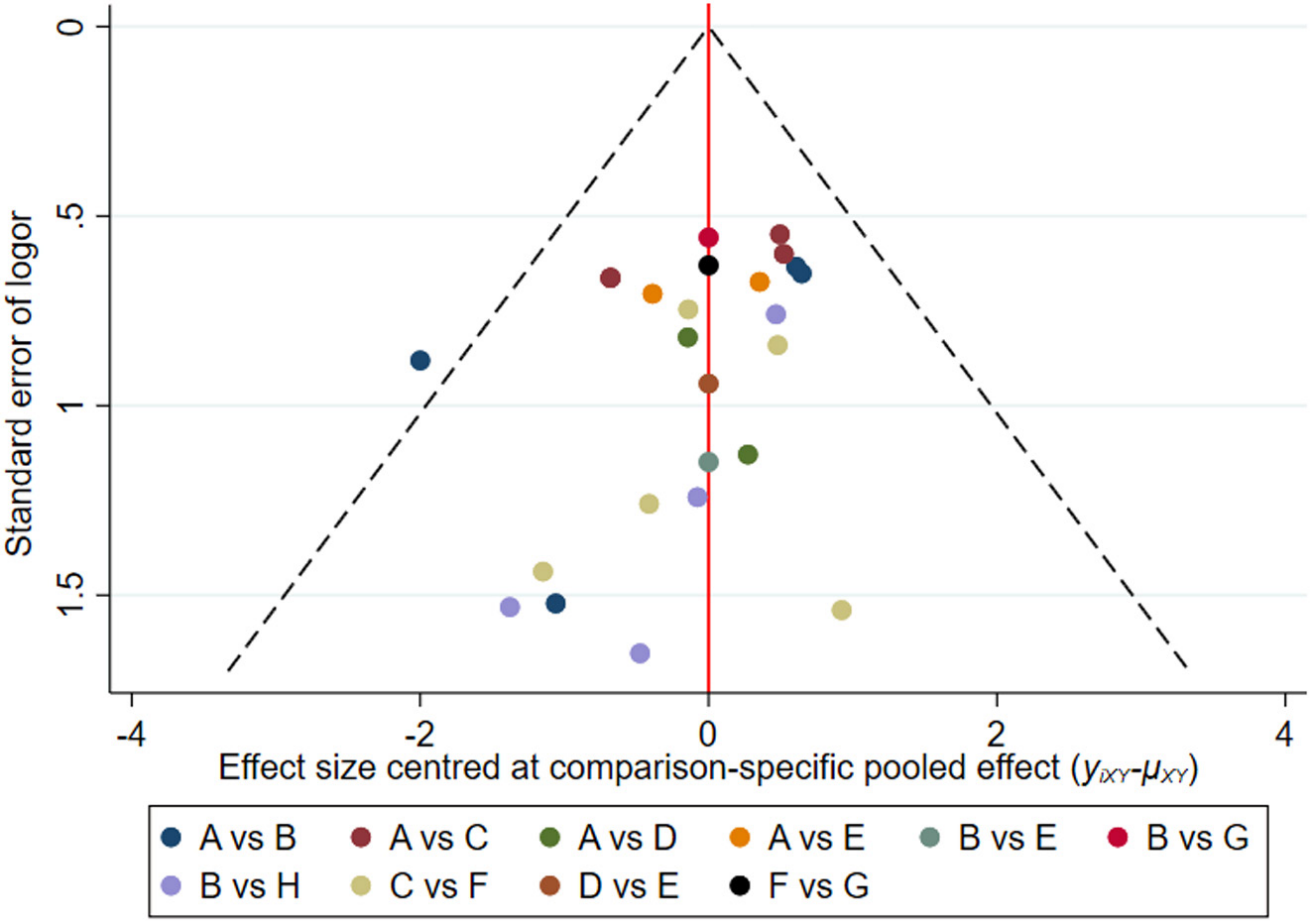
Comparison‐adjusted funnel plot of intraoperative obturator nerve reflex.

**FIGURE 14:**
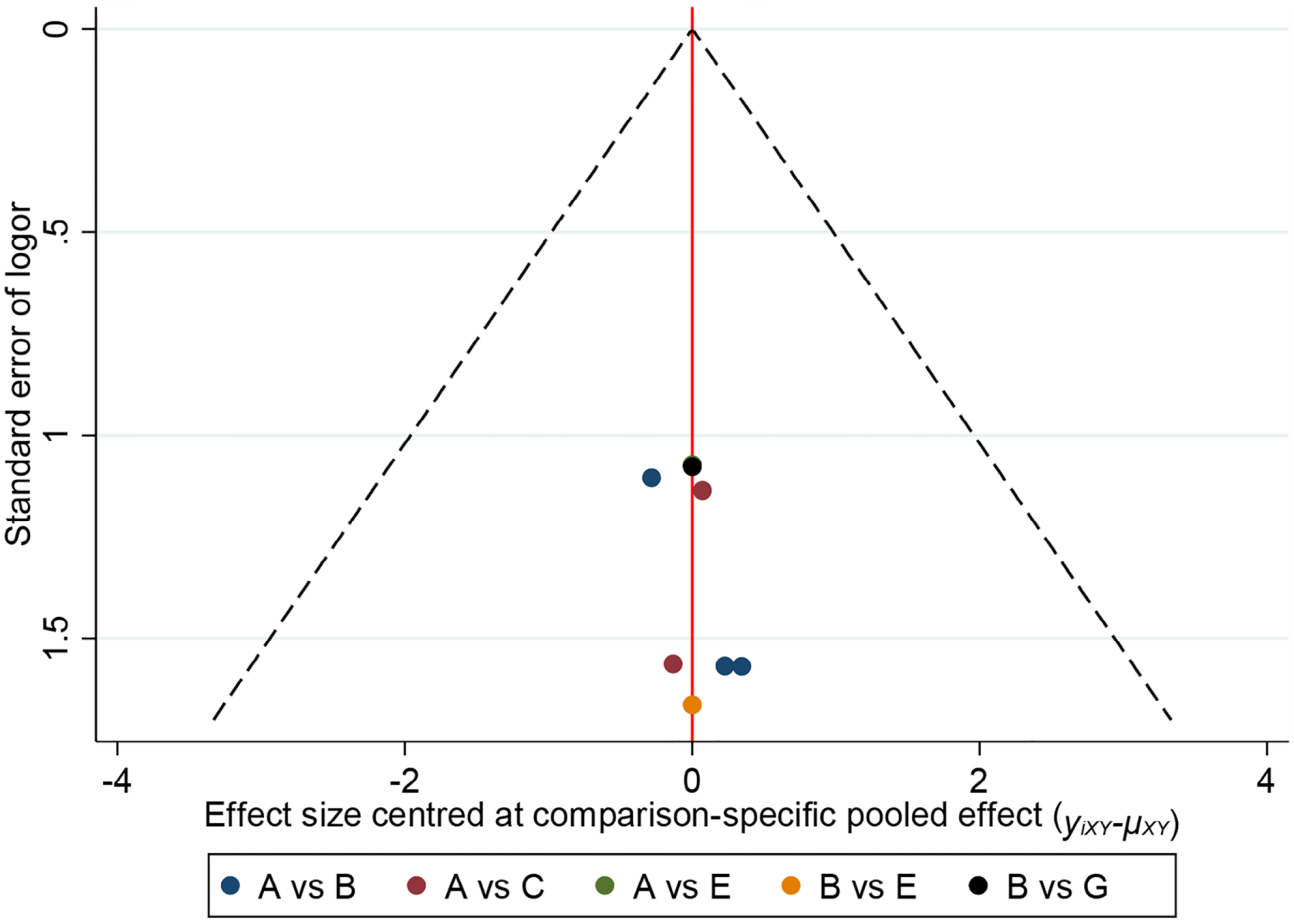
Comparison‐adjusted funnel plot of bladder perforation

Stata 14.2 software was used to draw the comparison‐adjusted funnel plot of obturator nerve reflex and bladder perforation. As shown in Figures 13 and 14, the distribution of the included studies was roughly symmetrical on both sides of the funnel plot, indicating that the studies may have a small sample effect or reporting bias.

## 4 DISCUSSION

Bladder cancer is the most commonly diagnosed malignancy of the urinary system, and its incidence is increasing due to a globally aging population.^32^ In addition to geographical factors and age, smoking can greatly elevate the risk of bladder cancer. Although the economic burden remains higher in developed countries, it is expected to increase in developing countries due to changing lifestyle and prolonged life expectancy.^33^ These trends will pose a major challenge to health systems in both developed and developing countries in the near future.

Non‐muscle invasive bladder cancer is routinely diagnosed and treated by transurethral resection of bladder tumor (TURBT) combined with postoperative intravesical instillation. Previous studies have shown that 46.8% of the bladder tumors are localized to the lateral wall of the bladder, which is adjacent to the obturator nerve.^34^ Therefore, during resection of lateral bladder wall tumors, the electrical current may stimulate the obturator nerve reflex with adverse consequences. For example, the contraction of the adductor muscle caused by stimulation of the obturator nerve can affect the depth of resection. A resection with a shallow depth cannot completely remove the tumor, resulting in recurrence after TURBT. On the other hand, deep resection may cause bladder perforation and extravesical spread of the tumor cells, eventually leading to incomplete tumor resection and bleeding.^35^ In addition, some patients may experience delayed bladder perforation and bleeding during postoperative intravesical instillation. According to previously reported data, obturator nerve reflex occurs in 55.3%–100% of the TURBT cases involving the lateral bladder wall, causing adverse intraoperative and postoperative effects.^34^ Intraoperative perforation and incomplete tumor resection caused by the obturator nerve reflex increase the risk of postoperative recurrence and worsen prognosis. The combination of spinal anesthesia with ONB can significantly reduce the occurrence of obturator nerve reflex, and mitigate the above complications. In addition, the development of ultrasound‐guided and neurostimulator‐guided ONB has greatly improved its success rate and reduced complications due to the high‐precision puncture.^7^

A previous meta‐analysis showed that spinal anesthesia combined with various ONB methods reduced the occurrence of obturator nerve reflex more effectively compared to spinal anesthesia alone.^36^ However, there is insufficient evidence regarding the efficacy of two or more ONB approaches during TURBT. We conducted a network meta‐analysis based on the Bayesian model to summarize studies that directly and indirectly compared the incidence of obturator nerve reflex and bladder perforation during TURBT in response to spinal anesthesia combined with different ONB methods. A total of 19 RCTs and 5 retrospective studies were included in the meta‐analysis, which evaluated the rates of obturator nerve reflex and bladder perforation during TURBT using spinal anesthesia and seven different ONBs.

DONB NS was superior to dONB US in preventing the obturator nerve reflex, and in fact ranked above all other ONB methods, followed by pONB US and pONB NS. However, pairwise comparison of the top three ONB methods did not yield statistically significant results. Furthermore, pONB US was the optimal approach in terms of preventing bladder perforation. The proximal ONB, also known as the classical pubic approach, aims at the main obturator nerve or its major branches. Sufficient local anesthetic ensures complete coverage of this nerve and can block the nerve to the maximum extent. The pubic tubercle is the clearest anatomical marker for this approach. However, in cases where the pubic tubercle is difficult to locate, such as in obese patients or in patients with blunt pubis, the needle cannot access the obturator nerve and may pass above the pubic ramus, causing damage to surrounding structures like the bladder, rectum, and spermatic cord.

Complete ONB can be achieved by ultrasound‐guided injection of large volumes of local anesthetic without confirmation by electrical stimulation. In addition, color Doppler flow imaging can reduce the incidence of vascular puncture during the operation. Generally, distal ONB has a higher success rate and fewer complications. However, due to the small size and deep location of the posterior branch of the obturator nerve, it is difficult to locate it precisely under ultrasound guidance. Ultrasound only allows visualization of the local injection and diffusion of the anesthetic between muscle layers.^37^ Therefore, routine anesthetization cannot achieve complete blockade of the obturator nerve with ultrasound. This is consistent with the greater efficacy of pONB US, pONB NS, and dONB NS + US compared to dONB US observed in our analysis. Thus, local blockade using a peripheral nerve stimulator remains the key to improving the efficacy of ONB,^38^ since it enhances the safety of the block by improving accuracy and reducing the dose of the local anesthetic. We also found that pONB US and dONB NS had superior effects compared to tONB. For the latter, the anesthetic is injected into the bladder wall near the path of the obturator nerve and the area surrounding the bladder tumor. This diffuse injection area lowers the efficacy of tONB.^37^

PONB US combined with spinal anesthesia is most likely the best approach to prevent bladder perforation and ranked second to only dONB NS in terms of preventing the obturator nerve reflex. The proximal approach targets both the common obturator nerve and its major branches, and a larger volume of local anesthetic ensures complete coverage of the nerve.^39^ Complete ONB can be achieved by ultrasound‐guided infiltration of sufficient local anesthetic in the absence of confirmation by electrical stimulation. In addition, color Doppler flow imaging can reduce the incidence of vascular injury during the operation.

### 4.1 Limitations

Five of the included studies did not mention the specific method of generating a random sequence, and only five studies reported that the random assignment process was conducted in a blinded manner. Therefore, a research design bias did exist to some extent. In addition, the different anesthetics and doses used for ONB across the studies may also have caused bias and increased the heterogeneity of the meta‐analysis. Although we identified spinal anesthesia combined with pONB US as the optimum approach for preventing bladder perforation, there are no relevant articles on spinal anesthesia combined with dONB NS, which precludes any comparison between the ultrasound‐ and nerve stimulator‐mediated techniques. Finally, since the number of included literatures is limited, subsequent multicenter, large‐sample clinical randomized controlled trials are needed to validate our findings.

## 5 CONCLUSIONS

Spinal anesthesia combined with dONB NS is the most optimal approach to prevent the obturator nerve reflex during TURBT. However, the appropriate ONB method eventually depends on factors such as the patient's general condition, tumor location, and doctor's proficiency in puncture techniques. The aim of ONB is to reduce the occurrence of obturator nerve reflex, reduce intraoperative bladder perforation, and achieve complete resection of the tumor.

## CONFLICT OF INTEREST*

All authors have completed the ICMJE uniform disclosure form. The authors have no conflicts of interest to declare.

## AUTHOR CONTRIBUTIONS

**Jinhao Wu:** Conceptualization (equal); data curation (equal); formal analysis (equal); methodology (equal); project administration (equal); software (equal); visualization (equal); writing – original draft (equal); writing – review and editing (equal). **Yafen Gao:** Conceptualization (equal); formal analysis (equal); methodology (equal); project administration (equal); software (equal); writing – original draft (equal); writing – review and editing (equal). **Zhiyong Xiong:** Data curation (equal); investigation (equal); writing – original draft (equal); writing – review and editing (equal). **Xiong Xiao:** Data curation (equal); writing – original draft (equal); writing – review and editing (equal). **Jun Yang:** Formal analysis (equal); project administration (equal); supervision (equal); validation (equal); writing – original draft (equal); writing – review and editing (equal). **Xiong Yang:** Data curation (equal); investigation (equal); supervision (equal); validation (equal); writing – original draft (equal); writing – review and editing (equal). **Yu Huang:** Conceptualization (equal); data curation (equal); formal analysis (equal); investigation (equal); software (equal); supervision (equal); validation (equal); visualization (equal); writing – original draft (equal); writing – review and editing (equal).

## Supporting information


Appendix S1
Appendix S2
Appendix S3
Appendix S4
Appendix S5

## Data Availability

The data that support the findings of this study are available from the corresponding author upon reasonable request.

## Endnote

* The authors are accountable for all aspects of the work in ensuring that questions related to the accuracy or integrity of any part of the work are appropriately investigated and resolved.
